# Improved gene delivery to human saphenous vein cells and tissue using a peptide-modified adenoviral vector

**DOI:** 10.1186/1479-0556-2-14

**Published:** 2004-10-08

**Authors:** Lorraine M Work, Paul N Reynolds, Andrew H Baker

**Affiliations:** 1 BHF Glasgow Cardiovascular Research Centre, Division of Cardiovascular & Medical Sciences, University of Glasgow, 44 Church Street, Glasgow, G11 6NT, UK; 2 Royal Adelaide Hospital Chest Clinic and Department of Medicine, University of Adelaide, Adelaide, South Australia, Australia

## Abstract

The establishment of efficient gene delivery to target human tissue is a major obstacle for transition of gene therapy from the pre-clinical phases to the clinic. The poor long-term patency rates for coronary artery bypass grafting (CABG) is a major clinical problem that lacks an effective and proven pharmacological intervention. Late vein graft failure occurs due to neointima formation and accelerated atherosclerosis. Since CABG allows a clinical window of opportunity to genetically modify vein *ex vivo *prior to grafting it represents an ideal opportunity to develop gene-based therapies. Adenoviral vectors have been frequently used for gene delivery to vein *ex vivo *and pre-clinical studies have shown effective blockade in neointima development by overexpression of candidate therapeutic genes. However, high titers of adenovirus are required to achieve sufficient gene delivery to provide therapeutic benefit. Improvement in the uptake of adenovirus into the vessel wall would therefore be of benefit. Here we determined the ability of an adenovirus serotype 5 vector genetically-engineered with the RGD-4C integrin targeting peptide inserted into the HI loop (Ad-RGD) to improve the transduction of human saphenous vein smooth muscle cells (HSVSMC), endothelial cells (HSVEC) and intact saphenous vein compared to a non-modified virus (Ad-CTL). We exposed each cell type to virus for 10, 30 or 60 mins and measured transgene at 24 h post infection. For both HSVSMC and HSVEC Ad-RGD mediated increased transduction, with the largest increases observed in HSVSMC. When the experiments were repeated with intact human saphenous vein (the ultimate clinical target for gene therapy), again Ad-RGD mediated higher levels of transduction, at all clinically relevant exposures times (10, 30 and 60 mins tissue:virus exposure). Our study demonstrates the ability of peptide-modified Ad vectors to improve transduction to human vein graft cells and tissue and has important implications for gene therapy for CABG.

## Text

Long term patency rates for CABG using autologous saphenous vein are poor, showing 1, 5 and 10 years post-CABG rates of 93%, 74% and 41%, respectively [1] and therefore represent a significant clinical problem. Long-term failures are due to neointima formation and superimposed atherosclerosis [2-4], a pathology that lacks a suitably efficient pharmacological therapy. Significant contributions of vascular smooth muscle cell (SMC) proliferation and migration have been documented [2-4]. Anti-proliferative strategies are in phase III clinical trial using decoy oligonucleotides to the transcription factor E2F, a strategy that has shown considerable promise pre-clinically [5,6] and also in early stage human trials [7]. We, and others have adopted the alternate strategy of gene therapy to prevent CABG failure [8]. CABG is an "ideal" clinical scenario for gene therapy since saphenous vein can be genetically modified *ex vivo *following leg harvesting and prior to coronary grafting. This unique "clinical window" has clear safety advantages over *in vivo *gene delivery since excess vector can be removed from the graft prior to coronary grafting. However, the clinical window is short (likely 10–60 minutes) and therefore necessitates the use of an efficient vector system for gene delivery. Adenoviral vectors have proven efficient for gene delivery in this context [9] although high titers are required to provide sufficient levels of gene delivery to achieve therapeutic gain using transgenes such as tissue inhibitor of metalloproteinases-3 [9], endothelial nitric oxide synthase [10] and p53 [11]. The latter study defined the rationale behind use of adenoviral vectors since long-term benefit on graft remodelling was shown at 3 months, even though the virus was only present for 2–4 weeks post grafting [11]. Any improvement in gene delivery above that mediated by adenoviral serotype 5 vectors would be very encouraging for clinical translation of pre-clinical therapies. To this end, a number of strategies have emerged including fiber switching (pseudotyping) and modification of adenovirus type 5 fibers with targeting peptides. Pseudotyping the fiber from adenovirus serotype 16, which binds CD46 [12], dramatically improves transduction to vascular cells including intact human saphenous vein allowing lower doses of vector to be used to achieve attractive levels of gene delivery to grafts *ex vivo *[13]. Likewise, gene delivery to vascular smooth muscle cells can be enhanced by incorporation of cell targeting peptides isolated by phage display into the HI loop of the adenovirus fiber [14], the preferred site for peptide insertion [15]. In the context of improved gene delivery mediated by the RGD-4C peptide, which was isolated by phage display and targets α_v _integrins [16], this has been shown for rabbit grafts [17] although the vast majority of data is based on gene delivery for cancer [18]. Since SMC show poor coxsackie and adenovirus receptor (CAR) availability [19], it is particularly relevant that the RGD-4C peptide may circumvent CAR deficiency on target cells to improve levels of transduction. In this study, we assess the ability of RGD-4C-modified adenovirus serotype 5 vectors to enhance gene delivery to human saphenous vein SMC and EC as well as to intact human saphenous vein *ex vivo*, the ultimate clinical target.

**Figure 1:**
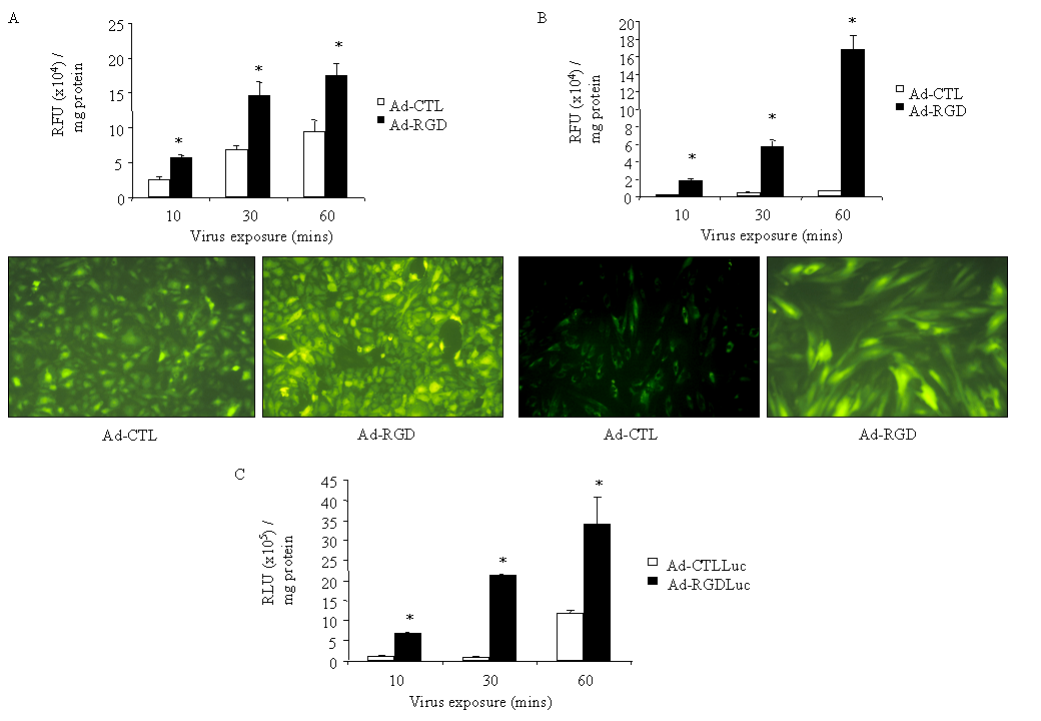
Transduction of saphenous vein cells and intact tissue. Ad-CTL and Ad-RGD expressing eGFP were incubated with (A) HSVEC and (B) HSVSMC for different times and gene expression quantified and normalised to protein. Representative fluorescent images are shown. (C) Intact human saphenous vein was incubated with luciferase-expressing vectors and expression quantified. *Indicates p < 0.05 vs Ad-CTL.

HSVEC were obtained by enzymatic collagenase digestion of human saphenous vein and maintained in endothelial cell complete media (TCS CellWorks, UK) supplemented with 20% (v/v) foetal calf serum (FCS; PAA laboratories, UK). HSVSMC were grown from medial explants from the same material and maintained in Dulbecco's modified Eagle's medium (DMEM) with 4500 mg/l glucose supplemented with 20% (v/v) FCS and 100 IU/ml penicillin, 100 μg/ml streptomycin and 2 mmol/l L-Glutamine. All cells were grown in a humidified atmosphere with 5% CO_2 _at 37°C. Cells were plated to reach 80% confluence 24 hours later. HSVEC or HSVSMC were infected in 96 well plates with increasing doses [plaque forming units (pfu) / cell] of Ad vectors for 10, 30, 60 mins at 37°C. The cells were washed twice in PBS and the media changed. 24 hours post-infection, the cells were again washed in PBS, lysed in PBS/0.2% Triton-X-100 and transduction quantified using the Wallac 1420 (Victor2) Multilabel Counter with recombinant eGFP (Clontech, Basingstoke, UK) as a standard. Reporter gene expression was normalised for total protein using the bicinchoninic acid (BCA) protein assay (Perbio, UK) with bovine serum albumin as standard, measured using a VICTOR2 plate reader. Exposure of HSVEC to Ad-CTL or Ad-RGD [50 plaque forming units (pfu) / cell] resulted in a time-dependent increase in the level of transduction (Figure 1A). At each time point studied (10, 30 or 60 mins), Ad-RGD mediated a significantly enhanced level of transgene expression compared to Ad-CTL (Figure 1A). Fluorescent microscopy demonstrated that control levels of infection with Ad-CTL were relatively high in HSVEC but further enhanced using the RGD-modified Ad (Figure 1A) at all time points tested. This is consistent with HSVEC expressing moderate CAR levels [14,19] allowing transduction of cells by Ad-CTL but the RGD-4C vector can further improve virus uptake. In HSVSMC, Ad-RGD again mediated a marked and significant enhancement in levels of transgene expression at all time points studied (Figure 1B). HSVSMC were much less permissive to non-modified Ad-CTL infection (Figure 1B), consistent with our previous observations [14], but enhanced with Ad-RGD to near 100% transduction in HSVSMC by fluorescence microscopy (Figure 1B). Again, this effect was evident at all virus:cell exposure time points – 10, 30 and 60 mins. For both HSVEC and SMC similar RGD-4C-mediated increases were observed with different viral doses (10 and 100 pfu/cell; not shown) thereby showing both time- and dose-dependence.

Based on the above we therefore assessed transduction in intact human saphenous vein. In order to quantify transgene expression accurately in tissue extracts we used luciferase-expressing viruses. Intact human saphenous veins were cleaned of surrounding connective tissue and cut into rings 3–4 mm in length. During preparation and infection, veins were maintained in wash medium (RPMI supplemented with 100 IU/ml penicillin, 100 μg/ml streptomycin and 2 mmol/l L-glutamine). Individual vein rings were incubated with Ad vectors for 10, 30 or 60 minutes (1 × 10^9 ^pfu / ring) before being washed twice in PBS and maintained in organ culture for 5 days. Rings were maintained in wash medium supplemented with 30% (v/v) FCS. Vein rings were snap frozen in liquid nitrogen and homogenised using a mortar and pestle for determination of reporter gene expression 5 days post-infection. *Ex vivo *homogenates were suspended in 100 μL reporter lysis buffer (RLB) and kept on ice for 1 hour before supernatants were analysed for luciferase expression using the Luciferase Assay System (Promega). 96 well plates were prepared using 10 μL/well of homogenate suspension diluted to a total volume of 100 μL with RLB. 100 μL of Luciferase Assay Reagent was added to each well and the plate immediately read for 10 seconds per well. Detection was achieved using a Wallac 1420 (VICTOR2) Multilabel Counter with recombinant luciferase (Promega) as a standard and normalised for total protein. Ad-RGDLuc mediated a time-dependent increase in the level of transgene expression that was evident at all exposure times studied – 10, 30 and 60 minutes (Figure 1C). This demonstrates that the RGD-4C-modification of Ad vectors can increase transduction to human saphenous vein, especially at short exposure times. The kinetics of virus binding in relation to time is therefore improved through the RGD-4C peptide and has direct implications for the design of gene therapy vectors for use in human CABG gene therapy procedures in the future. Although we have previously shown that non-modified Ad vectors transduce both endothelial and smooth muscle cells during graft gene delivery [9], and here show increased transduction of both cell types *in vitro *with RGD-modification, it will be important to fully define the uptake of the RGD-modified virus in the intact vein at the cellular level by immunotechniques. In broader terms, the design and tailoring of viruses for individual cardiovascular gene therapy applications is an important aspect of translation from pre-clinical to clinical gene therapy.

## Competing interests

The authors declare that they have no competing interests.

## Authors' contributions

LMW performed all isolated cell culture and vein transduction experiments. PNR produced the viruses and AHB supervised all work as principle investigator

The authors thank Nicola Britton and Margaret Cunningham for technical assistance. This work was supported by the Biotechnology & Biological Sciences Research Council (E17190 to A.H.B) and the British Heart Foundation (PG03/031 to A.H.B.)
